# Hypertrophic Cardiomyopathy With Evolution to a Mixed Hypertrophic Noncompaction Phenotype

**DOI:** 10.1016/j.jaccas.2026.107743

**Published:** 2026-04-05

**Authors:** Neelia E. Abadi, Christopher A. Abadi

**Affiliations:** a Larner College of Medicine, University of Vermont, Burlington, Vermont, USA; b Heart and Vascular Division, Southcoast Health, Fall River, Massachusetts, USA

**Keywords:** echocardiography, cardiomyopathy, genetics, left ventricle

## Abstract

**Background:**

Noncompaction cardiomyopathy (NCCM) and hypertrophic cardiomyopathy (HCM) are both attributed to pathogenic sarcomere variants. Although a mixed HCM-NCCM phenotype has been described, temporal expression is not well understood.

**Case Summary:**

We present a 28-year-old male with findings suggesting a mixed, transitional HCM-NCCM phenotype. The patient was diagnosed with HCM in adolescence, requiring septal myectomy at age 20. Subsequent echocardiograms demonstrate evolving predominant features of NCCM.

**Discussion:**

Although mixed HCM-NCCM phenotypes have been documented in a minority of patients, the concept of a transitional phenotype with progressive findings of noncompaction is unique. This case suggests that HCM may obscure initial evidence of noncompaction. Some cases of NCCM diagnosed later in life could represent a transitional phenotype. The management and prognostic implications of this overlap are not established.

**Take-Home Message:**

A mixed, transitional HCM-NCCM phenotype may be more prevalent than previously recognized, requiring further investigation into its clinical and prognostic significance.

## Visual Summary

**Figure:**
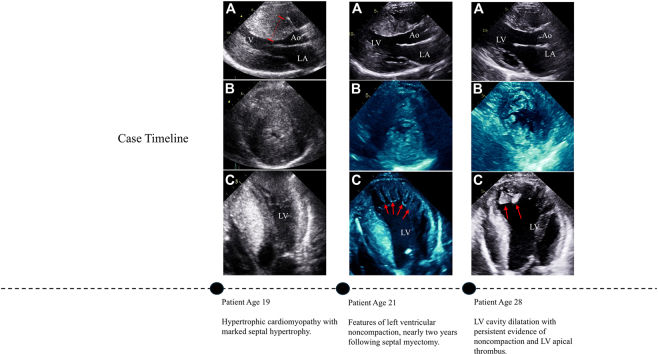

## History of Presentation

**Figure 1:**
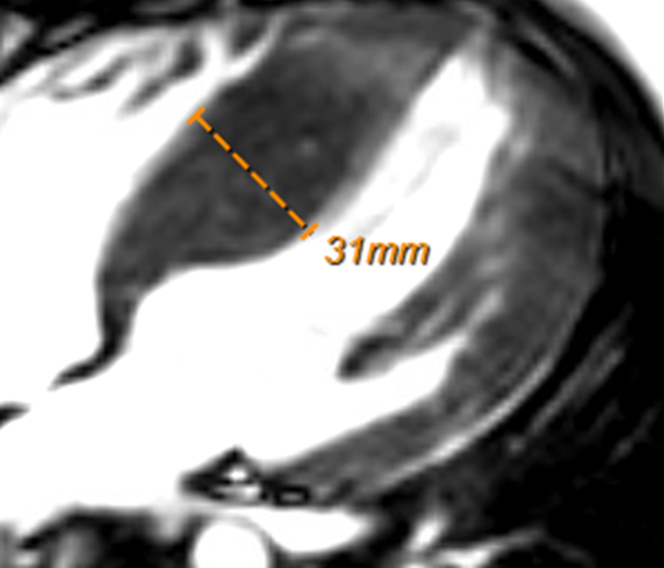
Initial Diagnosis of HCM The patient's cardiac magnetic resonance at age 13. Severe septal hypertrophy consistent with HCM. HCM = hypertrophic cardiomyopathy.

This patient was an otherwise healthy 13-year-old male with a family history of known hypertrophic cardiomyopathy (HCM) in his father. He was referred to pediatric cardiology for a screening echocardiogram, which revealed marked septal hypertrophy with septal thickness reported as 3.3 cm. Cardiac magnetic resonance (CMR) was thus performed, with left ventricle (LV) anteroseptal wall thickness reported as 3.5 cm (Figure 1) (3.1-cm 4-chamber view, limited images available), a posterior wall thickness of 0.8 cm, normal LV ejection fraction, and small foci of late gadolinium enhancement in the septum.

**Take-Home Messages**

• This case provides evidence that a mixed noncompaction phenotype can be masked and emerge with temporal separation in patients with HCM, underscoring the need for further investigation into the pathophysiology of this transitional relationship.
• Excluding noncompacted myocardium in patients with HCM can be challenging, and thus, close surveillance for evolution of noncompaction features is important, particularly given the varying risks and management considerations.

**Figure 2:**
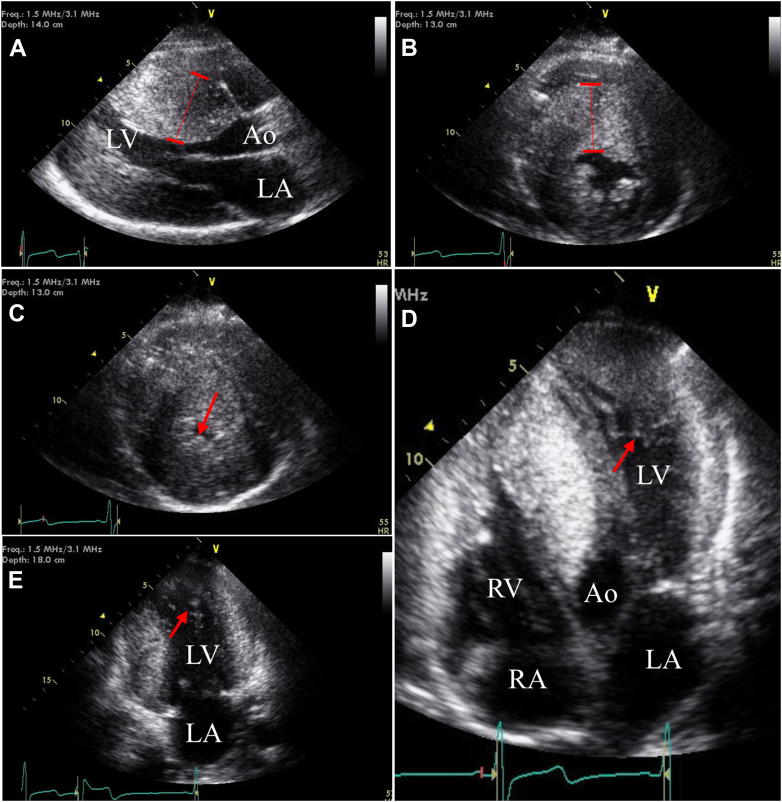
Hypertrophic Cardiomyopathy With Marked Asymmetric Septal Hypertrophy The patient's echocardiographic images at age 19. Parasternal long- (A) and short-axis (B) views of the LV in diastole with marked septal hypertrophy, measuring 3.5 cm. A short-axis view of the LV in systole with cavity obliteration (C). Apical 4- (D) and 2-chamber (E) views. On current review of imaging, evidence of trabeculation without recesses is present (red arrows in C to E). Ao = aorta; LA = left atrium; LV = left ventricle; RA = left atrium; RV = right ventricle.

**Figure 3:**
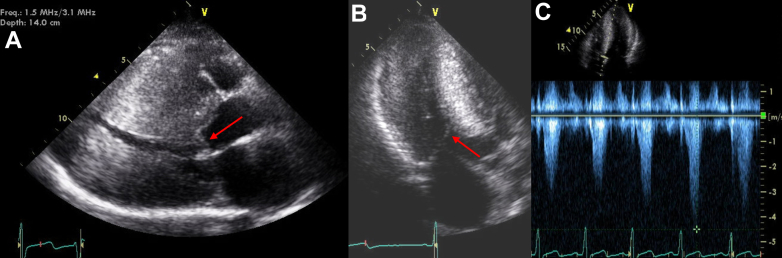
Systolic Anterior Motion of the Mitral Valve With Obstruction The patient's echocardiographic images at age 19. Parasternal long-axis (A) and apical 3-chamber (B) views in systole demonstrate septal contact of the mitral valve (red arrows). LV outflow gradient >80 mm Hg with Valsalva (C). LV = left ventricle.

A single-chamber implantable cardioverter-defibrillator (ICD) was placed due to marked septal hypertrophy (>3.0 cm), and he followed up annually with pediatric cardiology. At 18 years of age, he established care with adult cardiology and reported worsening exertional fatigue, lightheadedness, and dyspnea. A repeat echocardiogram at 19 years of age revealed marked septal hypertrophy (an LV end-diastolic thickness of 3.6 cm) with a small LV cavity—features consistent with HCM (Figure 2). Systolic anterior motion of the mitral valve with severe outflow obstruction, as well as a peak outflow gradient >80 mm Hg with exercise and Valsalva, was noted (Figure 3). LV ejection was normal. The managing team, including the team at an HCM center, did not observe trabeculation of the LV apex at this time. On current re-review of CMR and echocardiographic images, areas of trabeculation are observed, but intratrabecular recesses are not seen (Figure 2).

**Figure 4:**
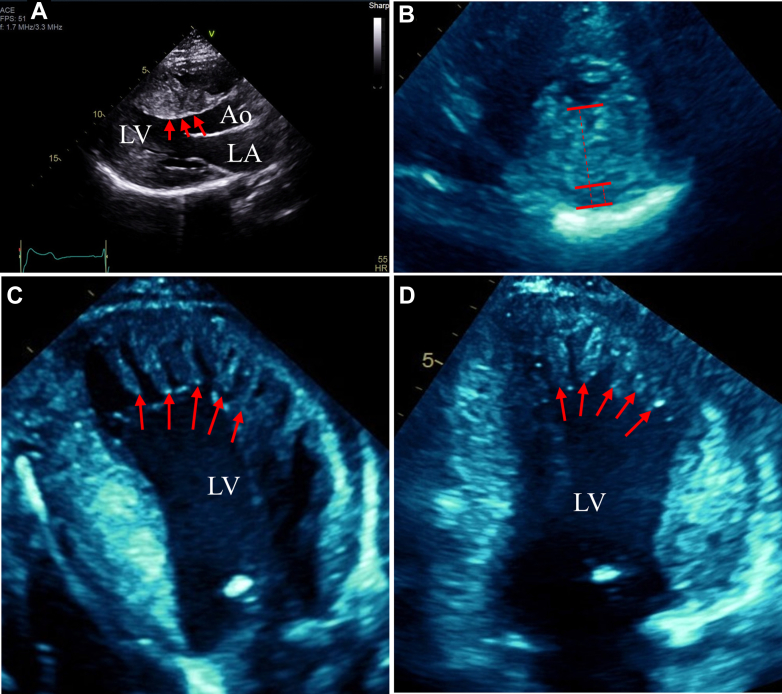
Noncompaction Pathology After Septal Myectomy The patient's echocardiographic images at age 21, nearly 2 years after septal myomectomy. The parasternal long-axis view in diastole demonstrates septal myomectomy results (red arrows) with significant reduction in septal wall thickness (A). The parasternal short-axis in end-systole (B) reflects a ratio of noncompacted to compacted myocardial thickness of >2:1, with noncompacted apical lateral and anteroapical myocardium, meeting much of the primary morphologic Jenni criteria for noncompaction.^1^ Apical 4- (C) and 2-chamber (D) views demonstrate prominent noncompacted muscle (red arrows) with large intertrabecular recesses. Ao = aorta; LA = left atrium; LV = left ventricle.

The patient subsequently underwent septal myomectomy with plication of the anterior mitral leaflet at an HCM center with an excellent result. He recovered appropriately and experienced symptomatic improvement. An echocardiogram nearly 2 years after surgery, at patient age 21, revealed no outflow obstruction; however, images now revealed evidence of LV hypertrabeculation and noncompacted muscle with large recesses. The ratio of noncompacted to compacted myocardial thickness was >2:1 when measured at end-systole in the short-axis view, with obvious noncompacted anteroapical and apical lateral myocardium, meeting much of the primary morphologic Jenni criteria for noncompaction (Figure 4).^1^ LV systolic function was normal. At this time, there also appeared to be some degree of regression of LV hypertrophy of the mid-anterior septum, separate from the region of myomectomy.

**Figure 5:**
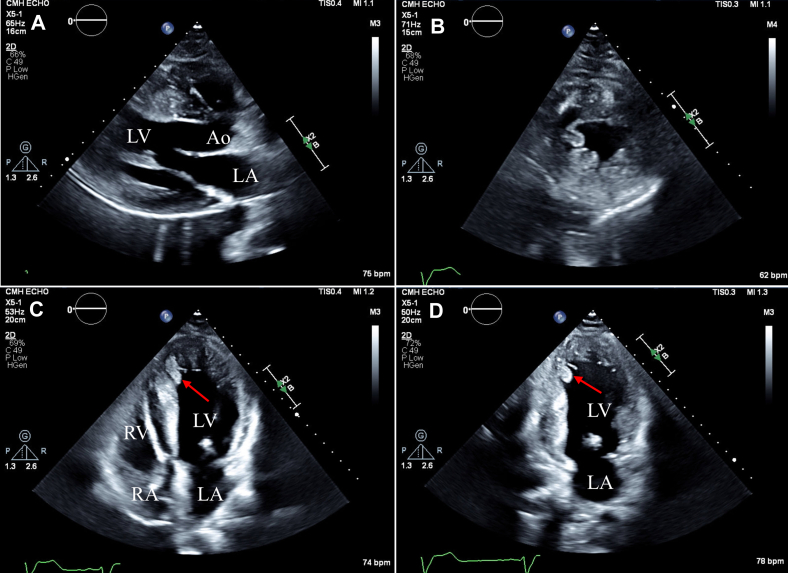
Progressive Features of Noncompaction With Apical Thrombus The patient's echocardiogram at the time of return to care at age 25. The parasternal long-axis view in diastole demonstrates LV cavity dilatation (A). The parasternal short-axis view demonstrates persistent evidence of noncompaction pathology (B). Apical 4- (C) and 2-chamber (D) views demonstrate apical dilation with a focal apical thrombus (red arrows). Ao = aorta; LA = left atrium; LV = left ventricle; RA = left atrium; RV = right ventricle.

The patient relocated shortly after this echocardiogram, before findings could be fully addressed, and was lost to follow-up. He returned to the area at 25 years of age and re-established with acute symptoms of dyspnea and fatigue. He was on no cardiac medications. An echocardiogram at this time revealed akinesis and dilation of the LV apex with an LV ejection fraction of 30% to 35%. Prominent noncompacted myocardium with large recesses and a focal apical thrombus were also noted (Figure 5).

**Figure 6:**
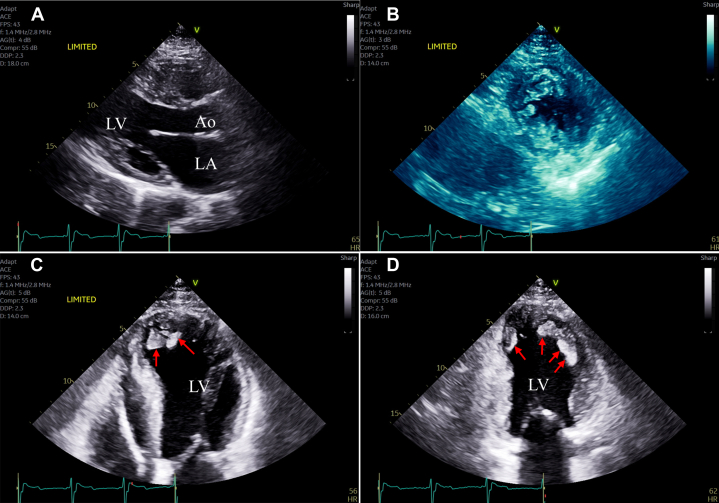
Complicated Features of Noncompaction With a More Extensive LV Thrombus The patient's echocardiographic images at age 28. The parasternal long-axis view in diastole with further LV cavity dilation and regression of hypertrophy (A). The parasternal short-axis view in systole with noncompaction (B). Apical 4- (C) and 2-chamber (D) views with an extensive thrombus (red arrows). Ao = aorta; LA = left atrium; LV = left ventricle.

He was started on guideline-directed medical therapy including anticoagulation with warfarin. He had unstable housing and ongoing challenges with compliance and therefore was transitioned to apixaban. Thrombus resolution was confirmed by echocardiography, and he remained stable from a symptom standpoint. However, he had inconsistent follow-up and self-discontinued all medications. He re-presented at 28 years of age with symptoms of congestive heart failure. Echocardiography was performed, now revealing further dilatation of the LV with an ejection fraction of 30% and multiple, large apical thrombi (Figure 6).

## Past Medical History

The patient had no significant past medical history before HCM diagnosis. Anxiety and alcohol use disorder developed in the patient's young adulthood.

## Differential Diagnosis

All initial testing in the patient's adolescence was consistent with HCM. However, on present review of imaging from ages 13 and 19 years, there is an indication of hypertrabeculation at the LV apex without recesses (Figure 2). After septal myomectomy, features of LV noncompaction with large intertrabecular recesses became visible on the echocardiogram. The full Jenni diagnostic criteria for noncompaction cardiomyopathy (NCCM) were not met due to HCM diagnosis.^1^ This pathology was not further investigated at this time as the patient was lost to follow-up. On re-presentation at age 25, symptoms of dyspnea and fatigue warranted further work-up. Differential diagnosis for these symptoms initially included heart failure with preserved ejection fraction secondary to HCM, new heart failure with reduced ejection fraction, recurrence of LV outflow tract obstruction, and mitral regurgitation. A repeat echocardiogram at age 25 confirmed the progression to complicated noncompaction features with LV dilatation, LV dysfunction, and apical thrombus. A mixed HCM-NCCM phenotype with a transition to predominant complicated noncompaction features is our final diagnosis.

## Investigations

Serial echocardiograms were obtained as described, with intermittent ICD checks. Gaps in care were noted due to unstable housing, patient relocation, and lack of insurance. ICD checks did reveal episodes of ventricular tachycardia, which responded to antitachycardia pacing. CMR was not repeated as an adult due to the presence of an ICD. Attempts were made to complete genetic testing, but compliance with appointments limited this patient’s care.

## Management

This patient has been treated with attempted guideline-directed medical therapy over the last several years, including a beta-blocker, an angiotensin II receptor blocker, a mineralocorticoid receptor antagonist, and apixaban. He follows up with electrophysiology for the management of his ICD.

## Outcome and Follow-Up

This patient was most recently back on metoprolol succinate, sacubitril/valsartan, apixaban, and low-dose aspirin. A follow-up echocardiogram is pending to reassess for resolution of apical thrombus. We hope to obtain genetic testing if the patient is agreeable.

## Discussion

NCCM is a rare and diagnostically challenging form of cardiomyopathy. Although there are no consensus criteria, the Jenni criteria are often recognized as a foundation for echocardiographic diagnosis. The condition is characterized by a thickened, 2-layer myocardium, prominent trabeculations, and deep intertrabecular recesses. Coexisting cardiac abnormalities must not be present to meet the Jenni criteria.^1^ Clinical profiles range from asymptomatic to arrhythmogenic, in association with systemic pathology.^2^ Symptomatic NCCM is associated with the risk of heart failure, ventricular arrhythmia, and thromboembolism.^3^^,^^4^ At the time of re-presentation at age 25, our patient had developed systolic dysfunction with apical thrombus and evidence for ventricular arrhythmia on ICD checks—both features of complicated NCCM.

HCM is an autosomal dominant condition that often has prominent involvement of the interventricular septum.^5^ Both HCM and NCCM are attributed to pathogenic variants in sarcomere genes, with some of the most notable including MYH7, MYBPC3, and TPM1.^2^^,^^6^^,^^7^ Although genetic testing has not been obtained in our patient, the combination of magnetic resonance imaging–confirmed HCM and a robust family history provides compelling evidence for an inherited sarcomere mutation. It is this shared genetic foundation that is the basis for a phenotypic overlap observed between NCCM and HCM. However, existing evidence reflects that only 4% of patients may have a true mixed phenotype—with noncompaction and features of classic HCM.^8^ Cases of isolated noncompaction have also been described in patients with family histories of HCM and identified sarcomere mutations.^5^ This clinical heterogeneity is reflective of variable gene expression within families.

In this case, noncompaction features were not apparent until after septal myomectomy and a change in LV volume. We also suspect myocardial fibrosis and/or reverse remodeling with regression of hypertrophy, most notably in the septum (separate from the myomectomy site) and the inferolateral wall.^9^ The patient subsequently developed LV dilation, LV dysfunction, and apical thrombus. On retrospective review of preoperative imaging, we suspect that noncompacted myocardium was present at initial presentation but was obscured by the marked concentric septal hypertrophy and the small LV cavity, with noncompacted muscle bands in contact. This pathology likely created a more confluent myocardial appearance without visible recesses. Limited visualization of the apical myocardium created further challenges. The expected embryologic basis of noncompaction and supporting pediatric data would reinforce the theory that preexisting noncompacted segments were present in our case but initially masked, complicating early detection.^10^

As LV cavity size and systolic dysfunction evolved, with further wall thickness remodeling, noncompaction features were able to predominate. Obvious recesses formed between noncompacted muscles, and then apical thrombi developed. Overall, this case suggests a mixed or transitional HCM-NCCM phenotype, with high-risk clinical features of each condition manifesting on different timelines.

Based on this case, investigation into whether a mixed, transitional HCM-NCCM phenotype is under-recognized would be appropriate. There are differences in management strategies between the 2 conditions, including approaches to sudden cardiac death risk stratification and indications for ICD placement. Patients with NCCM carry a higher risk of LV systolic dysfunction and intracavity thrombus formation in comparison with patients with HCM.^3^^,^^4^ Clarifying the degree to which these, and other risks, may be compounded in overlapping phenotypes would help inform prognosis and guide future management.

**Visual Summary:**
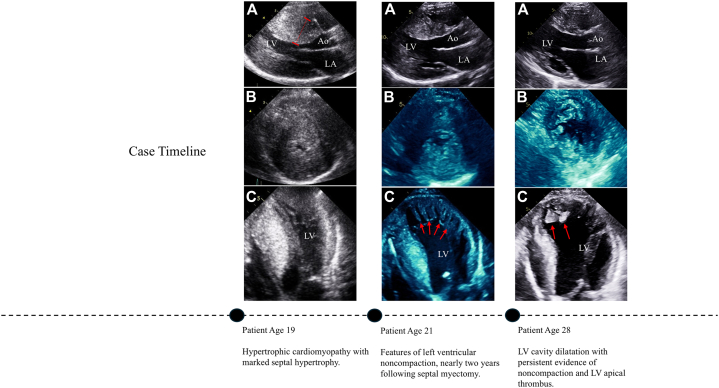
Case Timeline In this case, the patient was initially diagnosed with hypertrophic cardiomyopathy in adolescence. Transthoracic echocardiogram in the parasternal long-axis (A), parasternal short-axis (B), and apical 4-chamber (C) views illustrate a temporal transition to a mixed noncompaction phenotype with an evolving clinical risk. At age 19, images reflect classic features of HCM with marked septal hypertrophy (age 19: A-C). Following septal myectomy, at age 21, images demonstrate emerging features of noncompaction (age 21: A-C, noncompacted muscle identified with red arrows). At age 28, evolving features of complicated noncompaction with LV dysfunction and apical thrombus become apparent (age 28: A-C, apical thrombus identified with red arrows). Ao = aorta; LA = left atrium; LV = left ventricle.

## Conclusions

Both NCCM and HCM are recognized in association with sarcomere gene mutations. This case provides a unique lens through which we can understand how their mixed phenotype might evolve. To our knowledge, this longitudinal emergence of complicated noncompaction features from HCM is a unique finding, providing evidence to suggest that noncompaction in patients with HCM can become apparent on a clinically distinct timeline. This case also raises the question of whether some cases of NCCM diagnosed later in life may in fact be mixed or transitional, with HCM as the primary cardiomyopathy. Continued evaluation of a mixed or transitional phenotype is required to better characterize the clinical implications.

## Funding Support and Author Disclosures

The authors have reported that they have no relationships relevant to the contents of this paper to disclose.
